# Exosomes for mRNA delivery: a novel biotherapeutic strategy with hurdles and hope

**DOI:** 10.1186/s12896-021-00683-w

**Published:** 2021-03-10

**Authors:** Cynthia Aslan, Seyed Hossein Kiaie, Naime Majidi Zolbanin, Parisa Lotfinejad, Reihaneh Ramezani, Fatah Kashanchi, Reza Jafari

**Affiliations:** 1 Immunology Research Center, Tabriz University of Medical Sciences, Tabriz, Iran; 2 Department of Immunology, Faculty of Medicine, Tabriz University of Medical Sciences, Tabriz, Iran; 3 Nano Drug Delivery Research Center, Kermanshah University of Medical Sciences, Kermanshah, Iran; 4 Department of Pharmacology and Toxicology, School of Pharmacy, Urmia University of Medical Sciences, Urmia, Iran; 5 Department of Biomedical Sciences, Women Research Center, Alzahra University, Tehran, Iran; 6 School of Systems Biology, Laboratory of Molecular Virology, George Mason University, Manassas, VA USA; 7 Solid Tumor Research Center, Cellular and Molecular Medicine Research Institute, Urmia University of Medical Sciences, Shafa St, Ershad Blvd, P.O. BoX: 1138, Urmia, 57147 Iran

**Keywords:** mRNA therapy, Drug delivery, Exosomes, Extracellular vesicles, Immunogenicity, Toxicity

## Abstract

Over the past decade, therapeutic messenger RNAs (mRNAs) have emerged as a highly promising new class of drugs for protein replacement therapies. Due to the recent developments, the incorporation of modified nucleotides in synthetic mRNAs can lead to maximizing protein expression and reducing adverse immunogenicity. Despite these stunning improvements, mRNA therapy is limited by the need for the development of safe and efficient carriers to protect the mRNA integrity for in vivo applications. Recently, leading candidates for in vivo drug delivery vehicles are cell-derived exosomes, which have fewer immunogenic responses. In the current study, the key hurdles facing mRNA-based therapeutics, with an emphasis on recent strategies to overcoming its immunogenicity and instability, were highlighted. Then the immunogenicity and toxicity of exosomes derived from various cell sources were mentioned in detail. Finally, an overview of the recent strategies in using exosomes for mRNA delivery in the treatment of multiple diseases was stated.

## Background

Messenger RNA (mRNA), which is an intermediate molecule to transport genetic codes from DNA to ribosomes for protein expression has been suggested as a promising tool in novel therapeutic approaches for the treatment of several diseases and cancers [1, 2]. Exogenously delivered-mRNA has gained enormous attention due to its ability to encode any types of therapeutic proteins, including cytosolic, intra-mitochondrial, transmembrane, and secreted proteins [3]. In recent years, the potential therapy for various genetic defects has determined by a single gene, such as alpha-1 antitrypsin deficiency (AATD) [4], cystic fibrosis [5], and other monogenic disorders [6], as well as genetic diseases [7], brain diseases [8], infectious disease [9], cancers [10], etc. mRNA-based gene therapy is more advantageous, including no need any nuclear localization, and therefore no risk of genomic integration compared to classical gene therapy. Besides, it doesn’t alter the physiological state of the cell and also is not mutagenic due to its transient effect [6, 11]. Although naked mRNA hardly enters the cell, short plasma half-life, susceptible to cleavage by ribonucleases, and elicitation of innate immunity face difficulties in entering the cell [12].

Immunogenicity of therapeutic mRNA not only was improved by the incorporation of modified nucleotides [11] but also was significantly reduced higher efficiency combined with enhanced safety by the carrier with minimal immunogenicity, protection of mRNA degradation by nucleases, ability to pass through the phospholipid membrane, underlie efficient release from the cargo [10]. Moreover, an appropriate carrier enables repeated dosing without any cytotoxicity to achieve a sufficiently high quality of encoded protein, which will improve therapeutic efficiency [12]. Taken together, choosing the ideal carrier leads to higher efficacy combined with enhanced safety and decreased cytotoxicity; likewise, exosomes emerge the expected features due to the structural proximity with cellular components [13]. For the first time, exosomes, as natural carriers of mRNA- and microRNA-, were discovered in mast cells by Valadi et al. [14]. Exosomes were also detected as an important carrier of intracellular signaling in several other cells [15, 16]. Due to the biocompatibility nature of extracellular vesicles (EVs) with human cells, they successfully cross the cellular membrane and bypass drug delivery obstacles, including RNase degradation, endosomal accumulation, phagocytosis, multidrug resistance, cytotoxicity, and immunogenicity [17, 18].

In this review, we endeavor to summarize mRNA’s potential in the induction of an unwanted immune response. Besides, the current knowledge in the modification of mRNA to overcome its immunogenicity and then, the immunogenicity and toxicity profile of exosomes derived from various cell origins also be provided. Finally, we discuss the feasibility of engineering exosomes methods to utilize them as RNA drug delivery carriers.

## Hurdles of mRNA-based therapy

Even though mRNA was discovered in 1961 [19] for the first time, Malone et al. have displayed liposomes as mRNA carriers in 1989 [20]. In 1990, Wolff and colleagues described the idea to use using therapeutic mRNAs for producing specific proteins instead of classic gene therapy. However, due to the instability nature of mRNA molecules, they have not been considered therapeutic agents during that time. Besides the instability, immunogenicity was the other problem related to in vitro-transcribed mRNA (IVT) molecules [21].

### Immunogenicity of mRNA

One of the most critical hurdles to employ mRNAs as therapeutic agents is IVT mRNA’s immunogenicity [22]. Pattern recognition receptors (PRRs) are defined as specific structures that identify pathogen-associated molecules in infection [23]. Many cells recognize the single-strand RNA (SSR) and double-strand RNA (DSR) structures by PRRs, such as toll-like receptor (TLR) 3, 7 and, 8, which respond to SSRs and DSRs and induce the gene expression of pro-inflammatory cytokines (PICs) and type I interferons (IFNs) [24, 25]. The systemic delivery of unpurified IVT mRNAs can stimulate immune response and consequently induce the expression of (PICs) and (IFNs) [22].

In addition to TLRs, IVT mRNA can be identified by retinoic acid-inducible gene I (RIG-I) -like receptors (RLRs), which are cytosolic RNA helicases [26]. These cytosolic sensors, are primarily essential in innate immune and non-immune (epithelial) cells, such as RIG- I), melanoma differentiation-associated protein 5 (MDA5), and laboratory of genetics and physiology 2 (LGP2) [27–29]. Recognition of mRNA structures by TLR and RLR sensors, induces activation of transcription factors (TFs), including nuclear factor kappa B (NF-κB), IFN regulatory factor 3 (IRF3), and IRF7. Following activation of mentioned TFs, they bind to the gene promoter of IFN and lead to the induction of the expression of IFNs, in particular, IFN-α and IFN-β [30]. Moreover, the expression of PICs such as IL-6, IL-12, and *tumor necrosis factor-alpha* (TNF-α) is induced by NF-κB [26]. The stimulator of interferon genes (STING) is recently identified as an intracellular DNA sensor. Furthermore, STING has also been found to interact with RIG-I and the downstream adapter mitochondrial antiviral signaling protein (MAVS). As STING deletion results in impairment of RIG-I-mediated innate signaling, STING may play a role in anti-RNA virus defense [31]. Studies have shown that STING has not participated in dsRNA (poly IC) signaling, is mostly regulated by RLRs [32]. Nonetheless, loss of STING function renders mice highly susceptible to RNA virus infections, such as vesicular stomatitis virus (VSV), due to a decrease in type I IFN production in STING knockout cells infected with VSV, suggesting that STING may play a crucial role in maintaining homeostasis of the immune system [32, 33]. To overcome the stimulation of immune responses due to the presence of therapeutic mRNA, there are some strategies including using the synthetic modified mRNA [12]. The following studies explain whether nucleotide modification on mRNA could result in a reduction in its immunogenicity.

#### Synthesis of low immunogenicity mRNA with modified nucleotides

About a decade ago, innovative research by Karikó and Weissman et al. displayed that in vitro synthesis of mRNA molecule with the incorporation of modified nucleotides into the synthesized mRNA, results in more reduction of TLR-mediated immunogenicity and improves its translation and half-life [34]. To find more about nucleotide modification effect on immune response, several studies used different exogenous synthetic mRNA with variously modified nucleotides to investigate immune recognition and response by cells and organisms [35].

Durbin et al. used RIG-I-activating RNA ligand, the 106-nucleotide (nt) polyU/UC sequence derived from the 3′untranslated region (UTR) of the hepatitis C virus for discovering the immunosuppressive feature of different nucleotide modifications. Their results revealed that m6A, Ψ, m1Ψ, 5mC, 5-hydroxymethylcytidine (5hmC), 5-methoxycytidine (5moC), and 2′ fluorodeoxyribose modifications (2′ fluoro-deoxyuridine [2FdU] and 2′ fluoro-deoxycytidine [2FdC]) individually suppress RIG-I responses to the polyU/UC RNA ligand. Therefore, they displayed that RNAs containing modified nucleotides affect the initial stages of the RIG-I signaling pathway [36].

In 2015, Andries et al. found that the incorporation of the m1Ψ modification in combination with m5C on the mRNA leads to decreased cell cytotoxicity and innate immunogenicity because of the high potential of the modified mRNA to evade TLR3 activation and downstream innate immune signaling [37]. Moreover, Michel et al. developed a novel mRNA-based therapeutic method to resolve the single-gene defects, alpha-1-antitrypsin deficiency (AATD). They successfully delivered modified alpha-1-antitrypsin (AAT) encoding mRNA via lipofectamine agent into the different cell types. This study showed that delivery of dephosphorylated and modified mRNA induces just trivial expression of IFN-α, IFN-β, and TNF-α compared with the other types of modifications [4]. Moreover, according to the findings of Kormann et al., the combination of chemical modifications, 2-thiouridine and 5-methylcytosine, reduce the recognition of the modified mRNA via pattern recognition receptors such as TLR 3, 7, and 8 and cytosolic RIG-I in human peripheral blood mononuclear cells, lead to decreased immunogenicity with more stability in mice [38].

In contrast, Kauffman et al. reported that pseudouridine modification to mRNA had no effect on reduction of the serum levels of G-CSF, MCP-1, RANTES, and MIG as well as had no significant effect on mRNA immunogenicity in comparison to systematically delivered unmodified mRNA via liver-targeting lipid nanoparticles [39]. Consistent with this finding, Thess and colleagues reported that sequence-engineered mRNAs encoding erythropoietin (EPO) by incorporating the most GC-rich codon and made with unmodified nucleotides are not immunogenic, as evaluated by measuring inflammatory cytokines [1]. Additionally, a synthetic cap analog, such as the anti-reverse cap analogs (ARCA), can be used to further enhance translational efficiency and stability of mRNA and also reduce the immune activation [40]. In ARCA, the 3′-OH of the m7G moiety is replaced with a 3′-O-methyl group, which allows the cap analog incorporation in the proper orientation at the 5′-end during the IVT [41, 42]. Furthermore, circular RNAs (circRNAs) are a new class of RNAs with a covalently circular structure without a 3′ poly-A tail or a 5′ cap. Recently, they have attracted rising interest because of their prevalence and variety of possible biological roles [43]. Wesselhoeft et al. have shown that unmodified exogenous circRNA does not stimulate cellular RNA sensors and thereby evade an immune response in RIG-I and TLR competent cells and mice. They reported that unmodified circRNA has less immunogenicity than unmodified linear mRNA in vitro because of evasion of TLR sensing [44].

### Immunogenicity and toxicity of exosomes

**Fig. 1:**
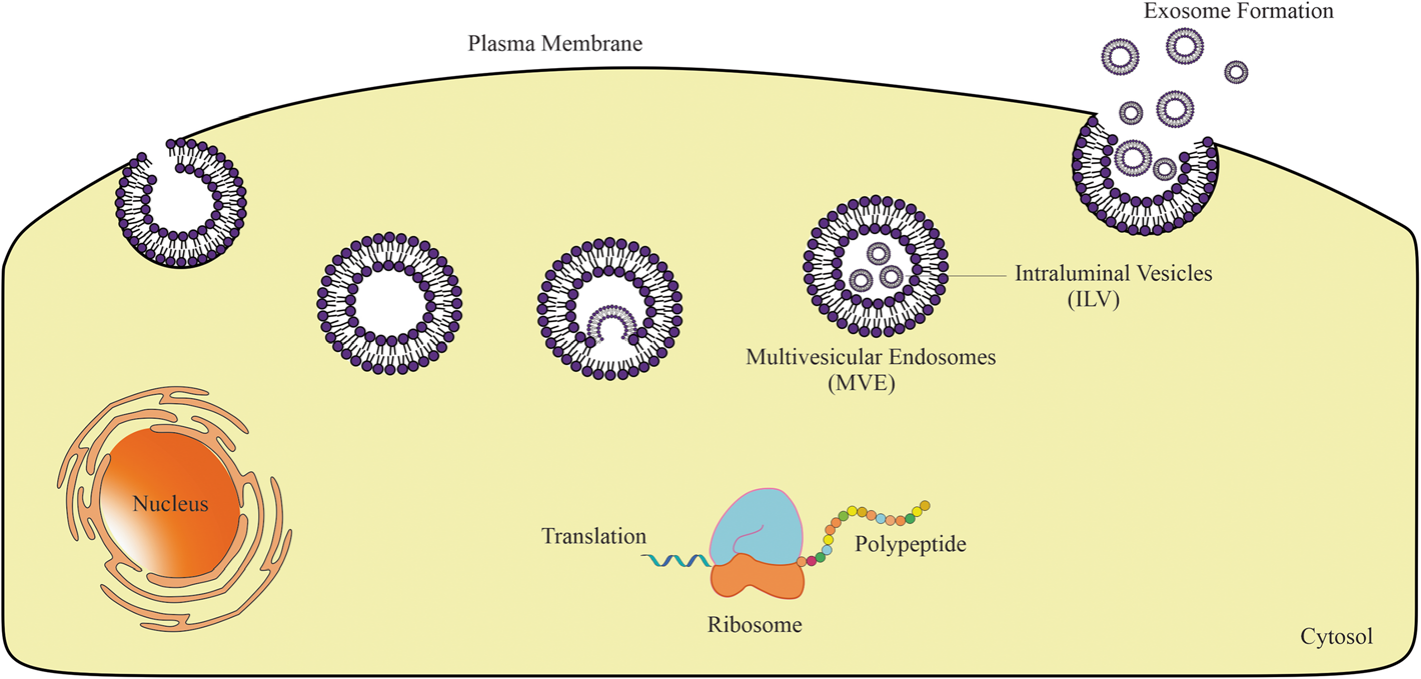
Exosome biogenesis: Exosomes are formed by producing MVB and ILV structures following endocytosis and inward budding of the MVB membrane

Despite recent advances in the development of nanomaterials that can carry drugs for cancer therapy, achieving an ideal drug delivery system while avoiding unacceptable toxicity, immunogenicity, and innumerable other side effects remains a crucial challenge. To overcome these obstacles, exosomes have been proposed as highly efficient to serve as a drug delivery device [45, 46]. Exosomes are nano-sized EVs (30–150 nm in diameter) which formed and released by almost all mammalian cells. Intraluminal vesicles (ILV) are formed by introversion of endosomal origin and endosomes, which are packed with these ILVs are entitled multivesicular endosomes (MVE) [47, 48]. The origination from the multivesicular body (MVB) and release into the extracellular matrix upon the fusion of MVB with the plasma membrane was depicted in Fig. 1.

Due to the inherent nanoscale dimensions and nature’s cellular product, these vehicles can escape phagocytic degradation, so they are naturally stable. They have intrinsic targeting properties based on their composition. Moreover, studies have shown that exosomes can cross the blood-brain barrier (BBB) [46, 49]. The biogenesis of EVs is an endogenous process that allows for two main strategies to load the EVs including pre-treatment of parental cells with agents of interest and then isolation of drug-loaded EVs from the conditioned medium, and actively or passively loading of isolated EVs with agents of interest [50].

To explore the characteristics and use of exosomes as a drug delivery system, a sufficient quantity of exosomes must be effectively isolated from different sources and must be free of cellular and molecular contaminations. Exosomes’ surface composition and cargo need to be carefully characterized to introduce the exosomes’ cargo repertoire and functions [51]. The therapeutic potential of exosomes depends on the ability of large-scale EVs production [52]. Despite the significant advances in EV isolation methods, currently, there is no distinct efficient technique for isolation of high purity exosomes due to the high biological sample complexity, EV heterogeneity, and intersection of the biological and physicochemical properties [53]. Existing exosome isolation techniques, obtain low exosomal yields and their large scale production for clinical researches and post-drug approval is expensive [54]. Through their formation, various cellular ingredients are wrapped in exosomes, which could potentially cause adverse side-effects in target cells such as toxicity and unwanted immune response. Future development of exosomes as therapeutics and drug delivery vehicles requires an in-depth understanding of their general safety and potential risks [55, 56]. The following studies represent the toxicity and immunogenicity profile of exosomes shed from various cell origins used as drug delivery vehicles.

### Challenges in exosome production

**Fig. 2:**
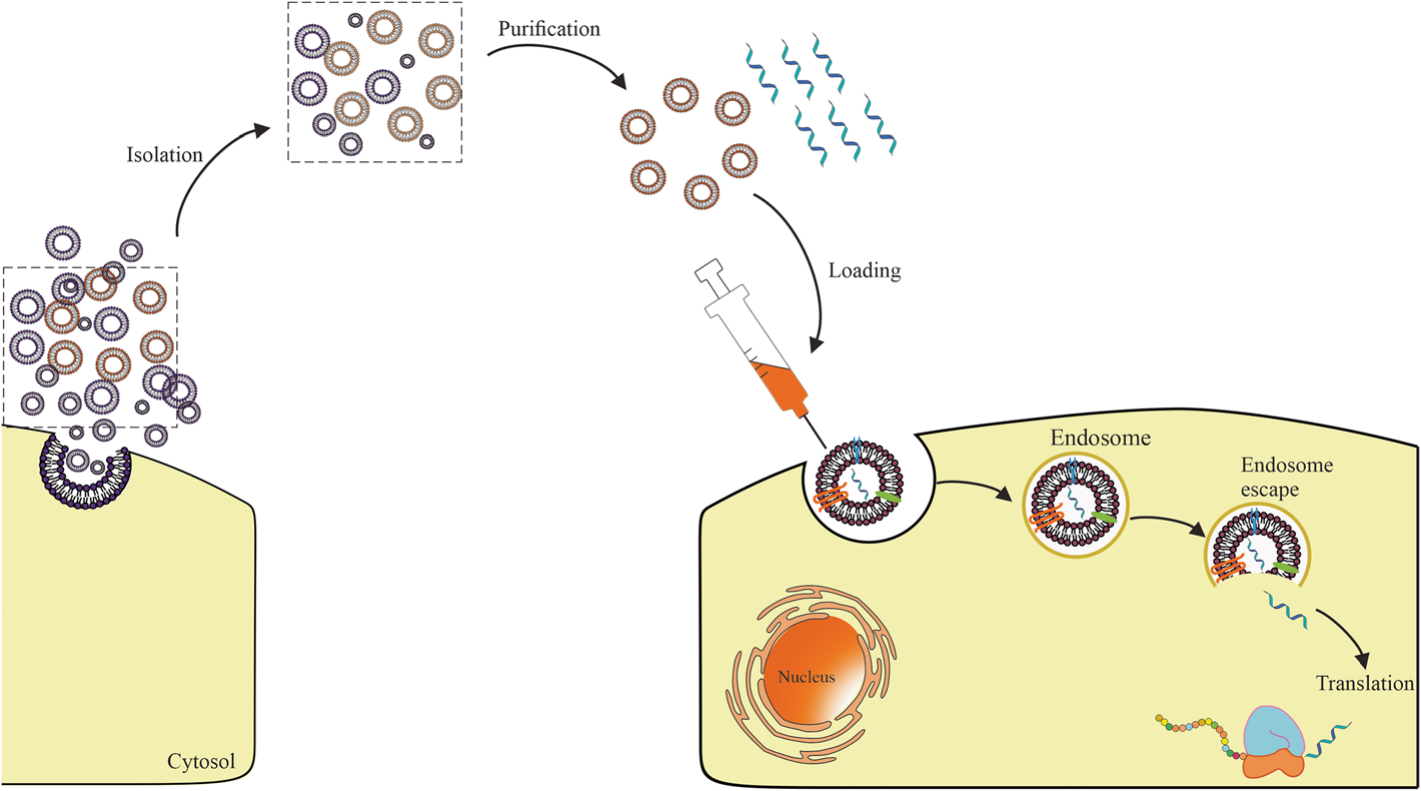
mRNA-encapsulated exosomes: Exosomes as carriers of mRNA enters the cell via endocytosis and escape from endosomes. Subsequently, mRNA is released into the cytoplasm and translated into proper peptides or proteins

Despite exosome keep away from phagocytosis or degradation by macrophages due to inherent small size and nature’s cellular product, prediction of long-term safety and therapeutic effect accounts for the ambiguous understanding of exosome nature and role complicated cutouts using of them. Furthermore, large-scale production for clinical trials shows the high cost and low quantity [46, 54, 57]. Sufficient translation of mRNA into the cytosol through vehicle was hindered due to its large molecular size, intrinsic instability, degradation by nucleases, and activation of the immune system [58]. Although chemical modification has been partially untangled some of these problems, a major obstacle is considered in the intracellular delivery of mRNA, which arising from the stabilization of mRNA stability under physiological exposure. Exosomes represent prodigious features including excellent permeation into physiological barriers, appropriate pharmacokinetic (PK), and tolerable immunological responses as an RNA carrier in comparison with other vehicles. However, exosomes indicate a suitable fitting strategy in small RNA (siRNA and miRNA) delivery and their yield for mRNA is low [59]. Recently challenge of inserting and release large quantities of mRNA in exosomes by enhancing the encapsulation through biological modification of cell sources and cellular nanoporation was resolved [18, 60, 61]. Therefore, it is essential to know and resolve the challenges in exosome production (such as sources, isolation and purification, and loading) for effective mRNA delivery (See Fig. 2).

The encapsulation efficiency of exosomes is hindered due to inherently packed with natural contents and short size range with the same number of encapsulated mRNA in each EVs [62, 63]. On the other hand, using good manufacturing practice (GMP) as a standardized manufacturing process in clinical trials to validate production and the therapeutic efficacy of exosomes is essential. In general, GMP-grade production refers to the type of cells, culture medium, cultivation system, and dissociation enzyme. In the following, purification includes a three-step process; cell debris filtration, concentrating condition medium (CM), and isolation from the concentrated CM. In this regard, understanding, and analysis of cell cultivation, purification, and quality control (QC) of exosomes. The main challenge in GMP-grade exosome production is to achieve appropriate QC. Furthermore, in the purification and characterization process, the development of GMP-grade animal-derived exosomes compared with plant-derived ones due to the less information was recommended [64, 65].

#### Exosome sources

##### Human embryonic kidney cells-derived exosomes

The human embryonic kidney (HEK) cell line (HEK293T) has been used EVs donor for a broad range of investigations, due to their high transfection efficiency, ease of growth, and capacity for high yield of EVs [66]. Li et al. pointed out that exosomes, as an ideal drug delivery tool, require cargo that causes minimal adverse effects and should have targeting ability. Their results indicated that the 293 T cell-derived exosomes might have the same features in common with the different tissues. Their resemblances at the membrane level improve exosome membrane fusion in these tissues. Moreover, they reported that few disease-related or cancer-related pathways were enriched in 293 T cell lines are regarded as appropriate in vivo drug delivery vehicles [67]. Furthermore, in another study, Rosas et al. showed that THP-1 and U937 monocytic cells, which represent a population essential in innate and adaptive immunity, internalized HEK293T-derived exosomes efficiently, and these exosomes did not exhibit a cytotoxic effect or alter phagocytic efficiency on THP-1 and U937 cell lines [56]. Additionally, a study by Zhu et al. reported that exosomes obtained from HEK293T cells, exert minimal toxicity and immunogenicity based on splenic immune cell composition or circulating cytokine levels in C57BL/6 mice following repeated dosing in 3 weeks [66]. HEK Expi293F cells also have desirable characteristics that make them promising exosome producers for clinical use [55]. A recent study by Saleh et al. reported the toxic and immunogenic potential of exosomes using HEK Expi293F cells as exosome donors. They treated the human hepatic cell line (HepG2) with Expi293F-derived exosomes to evaluate cell function, gene expression, and cytokine secretion of exosomes. As their results showed, no adverse effects were mediated in HepG2 cells after exosome treatment in 24 h. Moreover, they evaluated in vivo general toxicity of exosomes on BALB/c mice and reported minimal toxicity and immunogenicity, and pro-inflammatory cytokine response [55].

##### Bone marrow stem cells-derived exosomes

Bone marrow stem cells (BMSC)-derived exosomes have been proposed as a promising cell origin for producing clinical-grade exosomes for cellular therapy [68]. Mendt et al. generated engineered exosomes that have therapeutic potential to target oncogenic Kras (iExosomes) and they reported that repeated administration of mice to BMSC-derived iExosomes did not induce any detectable toxicity or harmful immune reactions in comparison to control mice, as evaluated by immune-typing of tissues, histopathological analyses, and secretion of PICs [68, 69].

##### Immature dendritic cell-derived exosomes

It has been shown, immature DCs (imDCs)-derived exosomes could weakly stimulate naïve T cells, probably due to lack of immune-stimulatory markers like CD86, CD40, major-histocompatibility-complex (MHC-I, and II) on their surface [70]. Based on this observation, Tian et al. have used mouse imDCs to generate exosomes for (DOX delivery to the tumor environment in BALB/c nude mice. In all, They suggested exosomes as attractive and ideal candidates for safe and efficient drug delivery for tumor-targeted therapy [71]. Moreover, Alvarez et al. demonstrated that imDCs-derived exosomes mediated siRNA delivery in vivo did not induce immune responses nor reveal any overt signs of toxicity [72].

##### Milk-derived exosomes

Milk has been proposed as a viable alternative source of exosomes due to its ease in scalability, safety, and biocompatibility [73] likewise Agrawal et al. have used bovine milk-derived exosomes for oral delivery of paclitaxel (PTX) which was termed (ExoPAC). As they measured systemic toxicity and immunogenicity of exosomes, ExoPAC, and PTX alone, they did not observe toxicity or any significant effect on the numbers of stem cells or immune cells (T cells, B cells, and neutrophils) populations by the exosomes or ExoPAC treatments. Furthermore, the number of T, B, and natural killer (NK) cells in the spleen did not change. Moreover, CD4 helper T cells and CD8 cytotoxic T cells were not altered by the exosomes or ExoPAC treatments [74]. Similarly, in another study, Munagala et al. indicated that milk-derived exosomes did not elicit any systemic toxic reactions or adverse immune response during short-term (1–6 h) or long-term (15 d) exposure in wild type rats; therefore they can act as a potential carrier for delivery of chemotherapeutic drugs [73].

##### Red blood cells-derived exosomes

In some cases, red blood cells (RBCs) have been used for producing exosomes for drug delivery. RBC-derived exosomes have several properties that are more suitable for clinical applications. Since RBCs are the most abundant cell type (84% of all cells) in the body and there is easy access to RBC-derived exosomes, either from maintained blood units at blood banks or even from the patients’ blood for allogeneic and autologous transfusion, respectively. RBCs release large-scale amounts about 1014 of exosomes during their maturation. Moreover, RBCs-derived exosomes are safe, because RBCs lack both nuclear and mitochondrial DNA, unlike EVs from other cell types. The successful compatible blood transfusion among people more develops the feasibility and clinical potential efficiency of RBC-derived exosomes for drug delivery [75]. In a study by Usman et al. exosomes were isolated from group O Rh-negative blood and successfully used for the delivery of RNA drugs to target a specific oncomiR gene in leukemia and breast cancer (BC) cells and they did not observe significant cytotoxicity in vitro or in vivo [76]. In another investigation, a pH-responsive superparamagnetic nanoparticles cluster-based strategy was designed to separate blood transferrin receptor-positive (TfR+) exosomes. These exosomes were used to deliver DOX on H22)in vitro(and 4 T1 cells)in vivo(. The results displayed high bio-safety of blood TfR+ exosomes along with the improved delivery of chemotherapeutic agents to the tumor environment [77].

#### Exosome isolation and purification

There are different techniques to isolate exosomes and the choice of the appropriate method depends on the type of the sample, for example, the source of exosome and downstream processes such as RNA and protein content analysis. These methods include ultracentrifugation, ultrafiltration, size exclusion chromatography (SEC), precipitation with polymers, and separation by affinity-based methods [47, 78, 79]. Recently, the devices based on microfluidic technology indicate promising advances for isolation and analysis of exosomes [80–82]. Therefore, look at the pros and cons of each method to find the qualified method of exosome purification is essential. So far, no method has been reported to purify the exosome that has all the desired features such as high efficiency and purity, simplicity, and no need for special and advanced equipment and facilities. Therefore, exosome purification is still one of the obstacles facing the application of these nanovesicles as carriers for biomaterials [83, 84].

##### Ultracentrifugation and ultrafiltration

Ultracentrifuge (UC) is considered a gold standard and the most common method for purification of the exosome, but the possibility of accumulation of vesicle masses due to centrifugal force is not negligible in this technique. Many factors, including force, rotor type, and solution viscosity, affect the result of exosome precipitation. However, this method is time-consuming and heavily instrument-dependent and is not suitable for separating exosomes from low-volume specimens such as clinical specimens and may be associated with some contaminant particles such as proteins and other vesicles. Moreover, the exosome structure may be lost due to a process called splat factor at high speed [78]. A density gradient ultracentrifuge (UC-DG) is sometimes used to purify and separate low-density exosomes from other vesicles and particles due to the density of exosomes, which is 1.13–1.19 gmL^− 1^. In the method that is based on the sucrose density gradient, the contaminants of proteins, lipoprotein, RNA, and large vesicles are removed, although it still indicates a time-consuming challenge [78, 85]. In ultrafiltration (UF), the exosomes are separated by size using filtration membranes, and the purification is based on the size and membrane molecular weight cut off. The use of filtration membranes does not allow vesicles and particles larger than the exosomes to pass through the filter, and the passage of the solution is accompanied by the removal of large vesicles. Although special materials are used in filtration membranes to reduce adhesion, some exosomes are still attached to the membrane and removed from subsequent analyzes. Following using the filters several times, their pores close and the pressure of passing solution affects the shape and integrity of the exosomes [79].

##### Size exclusion chromatography

In size exclusion chromatography (SEC), the separation is based on the size of isolated exosome particles due to different size distribution of microvesicles, proteins, and other particles and components in the biological materials. Despite SEC indicates a high degree of purity, the method efficiency is less than other methods. Although it does not take much time to separate each fraction, subsequent analyzes of each fraction to determine its exosome content are very time-consuming. Besides, there is a possibility of contamination in the column that should be considered [86, 87].

##### Polymer-based precipitation

The technology of precipitation of exosomes by polymers was introduced by System Biosciences (SBI) [88]. Although this technique is employed by commercial kits for exosome purification, it has been used for more than 50 years to isolate viruses from other macromolecules. Hebert was the first one who successfully concentrated plant viruses by polyethylene glycol (PEG) and sodium salt [89]. Other studies have also been reported on the isolation of viruses and bacteria using the same protocol [90, 91]. In this method, the exosomes, 60–150 nm in size, are trapped in a polymer network and precipitated with low-speed centrifuges; accordingly, kits such as Exo-Quick were launched [86]. However, the use of PEG or precipitation with polymers is very simple and does not require a special device, does not affect the particle properties and all steps of the purification process are done at physiological pH and in the absence of organic matter. In viral isolation studies with this method, the efficiency has been much more than ultracentrifugation but the drawback of this method is contamination with protein and non-vesicular components, which might be also precipitated along exosome. For this reason, several steps must be taken before and after separation, for example, the use of a 25 G-Sephadex column [47].

##### Immunomagnetic-based isolation

This method employs magnetic beads, which are coated with streptavidin and therefore bound to biotinylated antibodies. These antibodies detect biomarkers or antigens specific to exosomes, including CD63, CD9, and CD81, and thus separate only the exosome among other biomaterials. CD9 markers, for example, only detect and isolate serum exosomes. This method is used for special studies that require the separation of a special group of exosomes, and when a smaller volume of samples are available [92].

#### Exosome loading

Although it is quite feasible to load small RNAs (siRNA and miRNA) inside exosomes for oncogenic purposes in clinical specimens [93, 94], encapsulating mRNA in exosome remains a challenge. So far, several methods have been proposed to improve mRNA loading into exosomes, however, most of these methods were not successful, and further studies are needed to achieve more satisfactory results. In the following, the most used methods for mRNA loading into exosomes are mentioned.

##### Electroporation

Electroporation has long been known as the most rapid and efficient way to enter genetic material into a cell. In the method, mRNA can be entrapped in the exosome by using an electric field and creating small holes in the lipid bilayer of the exosome structure, just like the process that happens to a cell. This method is mostly used to load small RNAs such as siRNA and miRNA into exosomes [95], but there are some reliable reports that mRNA encapsulation in exosomes has been successful with this method [18, 96]. The main disadvantage of the method is the need to purify and separate the exosomes following the loading process [97]. To perform electroporation, the exosomes are necessary to be diluted in a special buffer, therefore they should be purified after the loading process. This can lead to the potential loss of part of the exosomes and reduces their quality.

##### Exosome-liposome hybrids

As we know, mRNA and DNA are easily loaded into liposomal structures, but liposomes cannot efficiently transfer these therapeutic cargos to target cells [98]. Unlike liposomes, exosomes are very efficient in delivering their cargo to the cell and releasing them due to their special transmembrane proteins for attaching to the cell and promoting endocytosis [99]. Lin Y. et al. reported that using the exosome-liposome hybrid, they could transfect target cells with plasmid DNA efficiently [99]. Although this method has not been used to load mRNA so far, it can be claimed that just as the same mechanism which DNA molecule loaded in the hybrid structure, mRNA can be entrapped too. Therefore, after binding mRNA to the cationic liposome, and then by incubating these liposomes with the exosomes, an exosome-liposome hybrid would be produced which can efficiently deliver mRNA to a cell [99]. Some liposome-mRNA compounds may have no interaction with the exosomes, so a separation step is required. According to the approaches, exosome-liposome hybrids have been shown to produce structures larger than 200 nm [99, 100], therefore ultrafiltration can be employed to separate them from a mixture of exosomes and liposomes whose size is much smaller.

##### Guidance of signature sequence

In this method, also called active loading, mRNA can be entrapped into the exosomes by employing some helper proteins. As we know, some proteins enable to bind to specific RNA sequences, Packaging of mRNA inside exosome can be easily guided through the fusion of the structural and specific proteins of exosome and creating an engineered cell [101, 102]. This method increases the efficiency of mRNA loading into the exosome [97].

##### Transfection of donor cells

The latest method of mRNA loading into exosomes is to transfect encoded DNA for this mRNA to the maternal cell, which is responsible for producing exosome (exosome-producing cell) [60]. In this method, 24 to 48 h after the transfection of the maternal cell, a culture medium containing exosomes released from the cell is collected and analyzed from the content of exosomal RNA, then the mRNA transcribed from a DNA vector is determined. Since the exosome-producing cell itself packs the desired mRNA into the exosome structure, we could introduce this technique as the most convenient method of mRNA loading into exosomes [61, 93, 96].

## The hope of using exosomes as a delivery vehicle to mRNA therapy

mRNA-based delivery technique faces similar obstacles, as well as other nucleic acids, include inefficient delivery [11]. Recent studies have shown increasing focus on, exosomes as promising carriers for mRNA drug delivery, due to their biocompatibility, bioavailability, and ability to cross BBB [10]. Theoretically, anything in the cell’s cytoplasm can be wrapped inside these small packages, including synthetic-mRNA from transfected parent cells [103]. Furthermore, isolated exosomes can be loaded with synthetic mRNA and chemotherapeutic drugs directly via conventional transfection methods [72]. Isolated exosomes can be passively transmitted throughout the body, but their ability to target distribution principally is associated with the surface-derived targeting molecules from parent cells [72]. Insertion of exosomes at destination cells occurs primarily by endocytosis, membrane fusion, or receptor-mediated internalization (Fig. 2) [104]. Recently, microneedle injector device indicates promising tool for transdermal delivery of exosomes due to painless and efficient dermal delivery in the skin for instance synthetic mRNA delivery with the high secretion of humanized Gaussia luciferase (hGLuc) protein [105] or mesenchymal stem cell (MSC)-derived exosomes with the low dosage in hair rehabilitation [106].

**Fig. 3:**
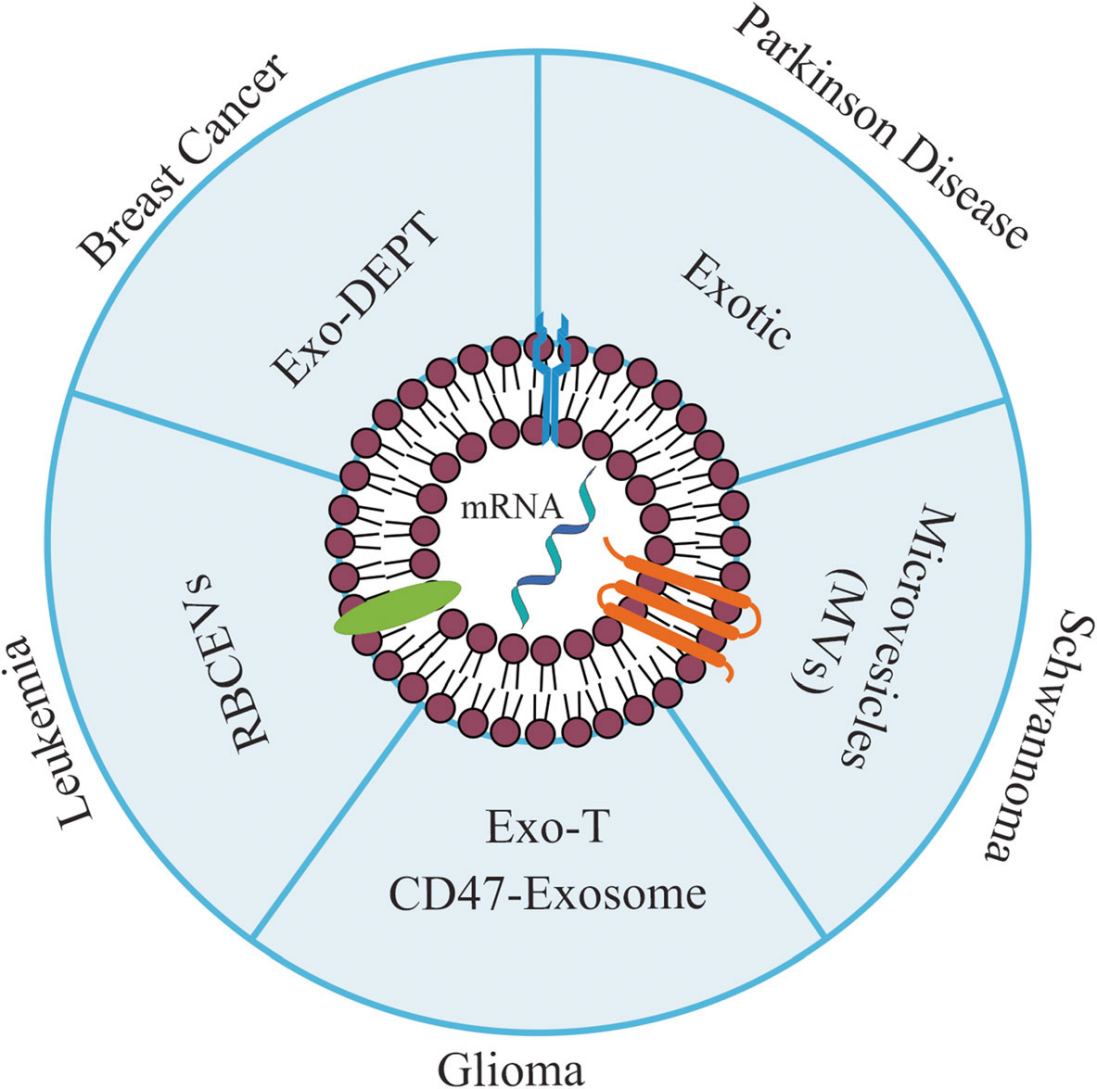
Exosome-mediated mRNA delivery for personalized medicine: different patient-derived cells such as a dendritic cell (DC), natural killer (NK) cell, or stem cell might be used as an exosome supply. mRNA is encapsulated into cell-derived exosomes and administrated to the patient

The following studies represent design strategies and recent advances in the exosome-based mRNA delivery systems to treat Parkinson’s disease, breast cancers, leukemia, glioma, and schwannoma (See Fig. 3).

### Parkinson’s disease

Since exosomes are regarded to have the potential to be used as RNA drug vehicles, Kojima et al. focused on the treatment of Parkinson’s disease and tried to deliver Catalase mRNA via designed exosomes to the brain. The most crucial reason is neuronal cell death, and the catalase-delivery is identified as a therapeutic approach to keep the neurons safe from oxidative damage [107]. They used a set of EXOsomal transfer into cells (EXOtic) devices in HEK-293 cells as exosome producer cells to improve exosome production, specific mRNA packaging, and release of the mRNA into the cytosol of recipient cells. They reported that therapeutic catalase mRNA delivered by designer exosomes, not only attenuated localized neuroinflammation induced by 6-OHDA in vitro and in vivo models of Parkinson’s disease but also rescued neuroinflammation caused by systemic injection of lipopolysaccharide (LPS) in vivo [60].

### Breast cancer

Wang et al. utilized exosomes because of their minimal immunogenicity, for delivery of HChrR6-encoding mRNA, to the HER2+ cells. HChrR6 as a bacterial enzyme can convert the prodrug (CNOB) into the drug (MCHB) in the tumors. They showed that exosomes loaded with HChrR6 mRNA (generated by transfection of cells with XPort/HChrR6 coding plasmid) and directed to the HER2 receptor (EXO-DEPTs), used in conjunction with CNOB, can specifically kill HER2+ cells, and cause near-complete growth arrest of BC in mice, but the tumors could not be eliminated [96]. Subsequently, HEK-293 cells were used as exosome donors, due to their minimal immunogenicity [96], although, in another study to further minimize the immune rejection, exosomes were generated by mice own dendritic cells [108]. Forterre et al. reported a lack of toxicity and suggested that the IVT EXO-DEPTs were instantly absorbed to HER2+ cells. This method had no significant changes in serum biochemistry components and whole blood hematology panels. Although, platelet counts (PLT), mean platelet volume (MPV), and total neutrophil counts were significantly altered. Since these variations were not so considerable and just has an impact on three hematological indexes, no critical bone marrow failure occurred. Histopathology evaluations of the liver, among untreated and treated mice displayed no differences (Fig. 3) [108].

### Leukemia

Usman et al. used human RBCs as exosome donors for RNA therapy. They treated acute myeloid leukemia (ALL) MOLM13 cells with RBC extracellular vesicles (RBCEVs) loaded with Cas9 mRNA and gRNA targeting the human mir-125b-2 locus. miR-125b is identified as an oncogenic microRNA in leukemia. The results demonstrated miR-125a and miR-125b expression were decreased about 90–98%, following a 2-day treatment. These results indicate that RBCEVs has the potential ability to deliver the CRISPR–Cas9 genome editing system efficiently into leukemia cells [18].

### Glioma

Recently, Yang et al. investigated whether exosomes could transfer a tumor suppressor gene called phosphatase and tensin homolog (PTEN) into the glioma brain tumors. In this study, glioma-targeting peptides were added to the N terminus of exosomal CD47, which enhanced the CD47-exosome (Exo-T) uptake in glioma cells. Following in vivo administration, Exo-Ts exhibit significant inhibition of tumor cell proliferation without any direct impact on other tissues and prolongation of animal survival [61]. This study displayed that the treatment with the CD-UPRT-enriched exosomes and the intraperitoneal administration of the prodrug 5-FC significantly reduced the tumor proliferation [109].

### Schwannoma

Mizrak et al. reported the study of engineered microvesicles (MVs) secreted by HEK-293 cells, loaded with suicide CD-UPRT mRNA or protein—for tumor therapy in vivo. They uncovered that delivery of the CD-UPRT mRNA/protein by MVs into the schwannomas via direct intratumoral administration resulted in regression of these tumors after systemic treatment with the prodrug 5-FC, which is converted within tumor cells to 5-FU [110].

## Conclusion

Taken together, as we have highlighted here mRNA offers outstanding advantages as a novel therapy over the gene therapy or substitution therapy, including induction of the expression of nearly all proteins, no need for nucleus phase for activity, and transient effect of mRNA, which enables the precise control of protein expression. On top of all that, developments made in mRNA technology in recent years, such as modification and purification methods, have made it possible to control the adverse immunogenicity and toxicity of mRNA and made it a unique therapeutic molecule. Further studies have now confirmed the potential of exosome as a novel mRNA delivery vehicle to increase the extracellular stability and made mRNA transfection more efficient as we highlighted before. But some critical challenges regarding exosomes are remaining to be solved toward the development of successful targeted drug delivery, including the yield of isolation of exosomes, component characterization, the targeting efficiency, sufficient drug loading capacity, and standardize exosome dosing. Furthermore, the question of which cell type to use as an exosome source for large-scale exosome production and safety issues remains to be answered. Collectively, further in vivo studies should aim at improving potency and reducing the toxicity of exosomes to explore these future directions moving towards therapeutic approaches in the coming years.

## Data Availability

Not applicable.

## Abbreviations

AAT: Alpha-1-antitrypsin
AATD: Alpha-1 antitrypsin deficiency
CD: Cytosine deaminase
Dox: Doxorubicin
EPO: Encoding erythropoietin
EXOtic: EXOsomal transfer into cells
ExoPAC: Exosomes for oral delivery of paclitaxel
EVs: Extracellular vesicles
5-FC: 5-fluorocytosine
2FdU: 2′ fluoro-deoxyuridine
5-FU: 5-fluorouracil
HepG2: Human hepatic cell line
5hmC: 5-hydroxymethylcytidine
IVT: In vitro-transcribed
mRNA: imDCs: Immature dendritic cells
LGP2: Laboratory of genetics and physiology 2
MDA5: Melanoma differentiation-associated protein 5
5moC: 5-methoxycytidine
MPV: Mean platelet volume
mRNA: messenger RNAs
MSCs: Mesenchymal stem cells
MVs: Microvesicles
PLT: Platelet counts
PRRs: Pattern recognition receptors
RBCs: Red blood cells
RBCEVs: RBC extracellular vesicles
RIG-1: Retinoic acid-inducible protein 1
RLRs: RIG-I-like receptors
TFs: Transcription factors
TLR: Toll-like receptor
UPRT: Uracil phosphoribosyltransferase
UTR: untranslated region
VEGF-A: Vascular endothelial growth factor-A
